# What determines the specificity of conflict adaptation? A review, critical analysis, and proposed synthesis

**DOI:** 10.3389/fpsyg.2014.01134

**Published:** 2014-10-08

**Authors:** Senne Braem, Elger L. Abrahamse, Wout Duthoo, Wim Notebaert

**Affiliations:** ^1^Department of Experimental Psychology, Ghent UniversityGhent, Belgium; ^2^Department of Experimental Clinical and Health Psychology, Ghent UniversityGhent, Belgium

**Keywords:** cognitive control, congruency sequence effect, task structure, associative learning

## Abstract

Over the past decade, many cognitive control researchers have studied to what extent adaptations to conflict are domain-general or rather specific, mostly by testing whether or not the congruency sequence effect (CSE) transfers across different conditions (e.g., conflict type, task sets, contexts, et cetera). The CSE refers to the observation that congruency effects in conflict tasks tend to be reduced following incongruent relative to following congruent trials, and is considered a prime measure of cognitive control. By investigating the transfer of this CSE across different conflict types, tasks, or contexts, researchers made several inferences about the scope of cognitive control. This method gained popularity during the last few years, spawning an interesting, yet seemingly inconsistent set of results. Consequently, these observations gave rise to a number of equally divergent theories about the determinants and scope of conflict adaptation. In this review, we offer a systematic overview of these past studies, as well as an evaluation of the theories that have been put forward to account for the results. Finally, we propose an integration of these various theoretical views in a unifying framework that centers on the role of context (dis)similarity. This framework allows us to generate new predictions about the relation between task or context similarity and the scope of cognitive control. Specifically, while most theories imply that increasing contextual differences will result in reduced transfer of the CSE, we propose that context similarity and across-context control follow a U-shaped function instead.

The study of cognitive control is generally concerned with how we adapt our information processing and action selection to constantly changing task environments and goals. Central to this research has been the study of cognitive conflict, where it is investigated how irrelevant information interferes with action selection by evoking conflicting responses. Previous work has convincingly demonstrated that humans (and other animals) have the ability to flexibly and rapidly adapt to such conflicting response activations, in order to carry out the rest of the task (or other related tasks that follow) more efficiently. In the current review we focus on the precise nature of such conflict adaptation processes by zooming in on empirical and theoretical work on the *congruency sequence effect* (CSE).

The CSE is a hypothesized marker of conflict adaptation and has served as an important research tool for investigating the scope of cognitive control. In the lab, the CSE is typically studied by means of a conflict task, such as the flanker, Simon, or Stroop task (Stroop, 1935; Simon and Rudell, 1967; Eriksen and Eriksen, 1974). In the Stroop task, for example, the participants' task is to respond to the ink color of the word, while ignoring the word's meaning. This way, congruent trials, where ink color and word meaning evoke similar responses (e.g., the word GREEN printed in green), and incongruent trials, where ink color and word meaning evoke different responses (e.g., the word RED printed in green), can be created. On incongruent trials, word meaning is believed to interfere with the processing of the ink color, thereby slowing down and occasionally preventing accurate responses. The difference between reaction times or response accuracies is then referred to as the congruency effect (or in this task, the Stroop effect). Whereas the congruency effect is assumed to reflect conflict in information processing, the CSE is typically taken as a proxy for how people adapt their behavior in response to this conflict. Specifically, the CSE concerns the observation that congruency effects tend to be reduced after an incongruent as compared to after a congruent trial. This effect was first observed by Gratton et al. (1992), and is also known as the Gratton or conflict adaptation effect.

A central issue in discussing the nature of conflict adaptation concerns its specificity. On the one hand, it is possible that conflict adaptation is characterized by domain-general boosts in attention that allows us to enhance overall performance. However, as we shall see below, empirical work has resulted in theorizing on more specific adaptation processes. The major tool in exploring such specificity then concerns the transfer of the CSE across various conditions. For example, if one experiences a conflict in a particular task A, to which extent will this influence the processing of cognitive conflict in a subsequent task B? The current paper reviews this type of transfer studies and will consist of three main sections. In a first part of this review, we will outline some of the most prominent theories on cognitive control in general, and the CSE in particular. Importantly, besides conflict adaptation theories, this will also include theories that ascribe the CSE to non-conflict based adaptation but still have something to say about specificity of the CSE as well. Next, we will offer a brief but comprehensive overview of the empirical work thus far that investigated the transfer of the CSE across conditions. These two sections serve as a state-of-the-art reference guide for future work on the specificity of cognitive control. From there, however, we will also re-evaluate and integrate the ongoing theories and ideas in light of these empirical studies. We will close the review by identifying some outstanding research questions and outline how we can validate or falsify these new hypotheses.

## Theories on the congruency sequence effect

In this section, we summarize what we believe to be the most important current theories on the specificity of cognitive control, and the CSE in particular. Although these theories are obviously not mutually exclusive, we will extract from each its core notion (see Table 1). This overview is meant to be comprehensive, but not exhaustive. Therefore, our description of theories will focus on, and hence often be restricted to, the hypotheses concerning the specificity of cognitive control, without offering the computational details. We deliberately opted to first provide a general overview of the different theories, as this will allow the reader to better frame and evaluate the empirical findings reviewed in the section to follow.

**Table 1:** **Brief description of theories and their view on the scope of conflict adaptation**.

**Key publication**	**Conflict adaptation theory**	**Determinants of the scope of adaptation**
Botvinick et al., 2001	Conflict monitoring	Task-relevant information
Egner, 2008	Multiple conflict-control loops	Conflict type
Hazeltine et al., 2011	Set-level control	Task structure or task set
Verguts and Notebaert, 2009	Adaptation-by-binding	Active representations during conflict
Hommel et al., 2004	Feature integration	Active features or event files
Schmidt, 2013	Contingency learning	Contingencies

The most prominent theory of the CSE is the conflict monitoring theory by Botvinick et al. (2001). In this theory, cognitive conflict is proposed to be registered by the anterior cingulate cortex (ACC), which monitors the environment for conflicting response tendencies. This quantifiable measure of conflict then acts as a warning signal that motivates people to increase task focus. Although computationally specific about how conflict detection can be modeled (i.e., by measuring the activity at the response level), the conflict monitoring theory is underspecified in terms of how subsequent control can be autonomously implemented. In the example of the Stroop task, it predicts that ink color detection would be facilitated following Stroop conflict. In a flanker task, however, conflict adaptation would lead to an enhanced processing of target (location) information, relative to flanker (location) information. In this sense, the conflict monitoring theory thus proposes that conflict leads to an enhanced focus on the task-relevant stimulus dimension. This implies that the CSE would be restricted to the enhancement of task-specific processes and therefore would not transfer to alternative tasks: The CSE will only be observed when the previous and current *task-relevant information* remain the same.

Egner (2008) offered a more detailed theory on the specificity of conflict adaptation by stressing that the conflict type is what limits the impact of conflict processing on the previous trial. Egner's proposal is inspired by the taxonomy of Kornblum et al. (1990), which allows to differentiate conflict types on the basis of their overlap between, for example, the relevant and irrelevant stimulus dimension (e.g., Stroop task), or between the response dimension and irrelevant stimulus dimension (e.g., Simon task). Using this taxonomy, Egner (2008) proposes the concept of multiple conflict-control loops whereby the detection of one conflict type (e.g., Stroop conflict) will and can only lead to the enhanced recruitment of resources in dealing with that specific type of conflict. Therefore, this theory suggests that (dis)similarity in *conflict type* is the crucial factor determining whether the CSE will transfer from one task to the other.

Hazeltine et al. (2011) stress the role of *task structure* or *task set* in determining the specificity of conflict adaptation (see also Akçay and Hazeltine, 2008). Specifically, Hazeltine and colleagues argue that not the relevant stimulus features *per se*, but rather the entire task set will influence how participants perceive the task, and subsequently determine the scope of conflict adaptation. According to Hazeltine and colleagues, CSEs reflect adjustments in task representations and are highly sensitive to salient or relevant task boundaries. Therefore, the degree to which participants will perceive the tasks as (dis)similar (i.e., the subjective *task set boundaries*) will determine whether or not the CSE can be observed across tasks.

In their adaptation by binding theory, Verguts and Notebaert (2008, 2009) offer a new computational model to explain how adaptations to conflict occur. Although adopting the conflict monitor for conflict detection as proposed by Botvinick et al. (2001), Verguts and Notebaert (2008) take a different approach when it comes to how conflict adaptation is ultimately implemented. Specifically, they argue that upon conflict detection, a Hebbian learning signal is sent throughout the brain that strengthens all ongoing and active representations. As the CSE is typically studied following correct trials only, active representations are usually task-relevant associations and representations—and these are thus predominantly strengthened, leading to increased control. This theory is consistent with the views of Hazeltine et al. (2011) in that it similarly proposes that task representations and their associated boundaries are strengthened following conflict. However, this model goes one step further as this process is indifferent to the precise nature or task-relevance of these features, focusing rather on all active representations. According to the adaptation by binding theory, then, *every feature that is active during conflict* and coincides with the activation of one or the other task set can codetermine to which extent a CSE will occur on the subsequent trial.

The major reason why the adaptation by binding theory of Verguts and Notebaert is characterized by high specificity relates to the notion that conflict adaptation derives from associative learning processes, rendering it intrinsically bound to the overall set of representations that are active during a particular event, including both task-relevant and -irrelevant information (i.e., the overall context).

Interestingly, a number of theories have been proposed that understand the CSE not so much as a cognitive control phenomenon (i.e., it does not entail conflict-based adaptation), but rather as a direct consequence of specific episodic memory processes. First, Hommel and colleagues propose that CSEs reflect feature integration processes (Mayr et al., 2003; Hommel, 2004; Hommel et al., 2004; Nieuwenhuis et al., 2006). That is, on each trial all the available stimulus and response features are bound together into a so-called event file. However, when some of the features from the previous trial are reused in a new combination (i.e., partial feature repetition), this will require a breakdown of the event file that was formed on the previous trial, and this takes time. Partial feature repetitions taking more time than full repetitions or full alternations of feature sets can produce a similar behavioral pattern as underlies the CSE. Second, Schmidt and colleagues have argued that learning the contingencies between specific stimulus and response features can also lead to this behavioral pattern as especially congruency repetitions are benefiting from such contingency learning processes (Schmidt and De Houwer, 2011). It requires no detailed elaboration that both these episodic memory accounts predict very context-specific effects of the previous trial on the next: *every active feature* or *relevant contingency* can co-determine whether a CSE would be observed across conditions. Importantly, these two accounts, in contrast to the four before-mentioned accounts, do not see congruency identity of the previous trial as an important determinant for CSEs to occur.

## Transfer of the congruency sequence effect

In the previous section, we briefly sketched the stance that theories take on the specificity of the CSE. Here, we will offer an overview of the published empirical work that is relevant to this issue. Complementing our approach to the section above, where we discussed the theories without the data, we will now try to provide a theory-neutral description of the available data. As the scope of the CSE has been a popular topic in recent years, a substantial number of studies has contributed to the discussion of what determines its specificity. We will structure the discussion of these studies based on which research question they tried to tackle (see also Table 2). Specifically, we will first discuss all studies that tested the specificity of the CSE by investigating the potential for transfer across different types of conflict. Second, we will discuss a small set of studies that investigated transfer across conflict dimensions (e.g., vertical vs. horizontal Simon task). Last, we will discuss studies that looked at the impact of specific task parameters (i.e., response or stimuli sets) or more contextual task-irrelevant factors, respectively.

**Table 2:** **Studies investigating the scope of cognitive control, using the congruency sequence effect (CSE)**.

**Authors**	**Conflict tasks**	**Method**	**Findings: specific or global?**
**CONFLICT TYPE**			
Akçay and Hazeltine, 2011	Simon and flanker	A factorial combination of a Simon and a flanker task	*Specific*. CSE was observed within, but not across conflict type
Boy et al., 2010	Flanker and prime-target	A factorial combination of a Simon and a spatial prime-target task	*Specific*. CSE was observed within, but not across conflict task
Egner et al., 2007	Color Stroop and Simon	A factorial combination of a Simon and a color Stroop task	*Specific*. CSE was observed within, but not across conflict task
Fernandez-Duque and Knight, 2008	Number Stroop and Flanker or color Stroop	Performance on a number Stroop task was investigated as a function of previously (cued) flanker or word Stroop congruency	*Global*. CSE was observed across conflict tasks. Notably, the congruency identity of the previous trial was always cued
Forster and Cho, 2014	Simon and Stroop	A Simon and Stroop task with shared response sets were presented in fixed or mixed blocks	*Specific*. CSE was observed within, but not across conflict task
Freitas et al., 2007, Experiments 2 and 3	Flanker and color Stroop or spatial Stroop	An arrow flanker task was intermixed with either a color word Stroop task (Experiment 2), or a spatial Stroop task (Experiment 3)	*Global*. CSE was observed across conflict tasks
Freitas and Clark, 2014, Experiments 2 and 3	Stroop-trajectory, Spatial Stroop, flanker, and Simon	Two different Spatial Stroop tasks were intermixed with a flanker task (Experiment 2) and a newly developed Stroop-trajectory task was intermixed with a flanker and Simon task (Experiment 3)	*Global and specific*. CSE was observed across conflict tasks, except across the Simon and Stroop-trajectory task
Funes et al., 2010a	Spatial Stroop and Simon	A Spatial Stroop task was intermixed with a Simon task	*Specific*. CSE was observed within, but not across conflict tasks
Funes et al., 2010b, Experiments 1 and 2	Spatial Stroop and Flanker or Simon	A Spatial Stroop task was intermixed with a Flanker (Experiment 1) or a Simon (Experiment 2) task	*Specific*. CSE was observed within, but not across conflict tasks
Kan et al., 2013	Color Stroop and sentence processing or perceptual ambiguity	Stroop trials were intermixed with a sentence processing task in a first experiment, and with a perceptual ambiguity task in a second experiment	*Global*. CSE was observed from the sentence processing task, as well as the perceptual ambiguity task, to the Stroop task
Kleiman et al., 2014	Flanker task and a gender flanker task or race priming task	The influence of flanker congruency on stereotypical biases was investigated combining a letter flanker task with a gender flanker task (Experiment 1) or race sequential priming task (Experiment 2)	*Global*. Stereotypical biases were observed following flanker congruent trials, indicated by a CSE from the flanker task to both the gender flanker task, and the race priming task
Kim et al., 2012	Color Stroop and arrow Stroop	A factorial combination of a color and arrow Stroop task	*Specific*. CSE was observed within, but not across conflict type
Kunde and Stöcker, 2002	Spatial and temporal Simon	A factorial combination of a temporal and spatial Simon task	*Specific*. CSE was not observed across conflict type, but, importantly, also not within-conflict type for the temporal Simon task
Kunde and Wühr, 2006, Experiment 2	Simon and prime-target	A factorial combination of a Simon and a spatial prime-target task	*Global and specific*. CSE was observed across conflict tasks, but was smaller across than within conflict type
**CONFLICT TYPE**			
Kunde et al., 2012	Simon and affective interference	A factorial combination of an affective interference and a Simon task was used where the interference was either of a different type (Experiment 1) or the same type (Experiment 2)	*Specific*. CSE was observed within the conflict types but not across the conflict types in both experiments
Rünger et al., 2010	Flanker and number Stroop	Performance on a number Stroop task was investigated as a function of previously (cued) flanker congruency	*Specific*. CSE was not observed across tasks. This study was set up as a replication study and reported as a replication failure of Fernandez-Duque and Knight (2008)'s Experiment 4
Schlaghecken et al., 2011	Simon and prime-target	A factorial combination of a Simon and a spatial prime-target task	*Specific*. CSE was observed within, but not across conflict type
Verbruggen et al., 2005	Spatial Stroop and Simon	A Simon task was intermixed with a Spatial Stroop task	*Specific*. CSE was observed within, but not across conflict type
Wendt et al., 2006	Simon and flanker or Stroop	A factorial combination of a Simon and a flanker task (Experiment 2A) or a Simon and a Stroop task (Experiment 2B) was used	*Specific*. CSE was observed within, but not across conflict type
Wühr et al., 2014	Simon and Stroop	A manual Simon and verbal Stroop task (Experiment 3) were intermixed	*Specific* CSE was observed within, but not across conflict type
**CONFLICT DIMENSIONS**			
Cho et al., 2009	Stimulus response compatibility task	A stimulus-response compatibility task was used where each trial was preceded by a cue denoting an either compatible or incompatible response mapping along a horizontal or vertical dimension	*Global*. In four experiments, a CSE was observed across dimensions
Freitas et al., 2007, Experiment 1	Flanker	An arrow flanker task was administered that was oriented on either a horizontal or vertical dimension	*Global*. CSE was observed across dimensions
Freitas and Clark, 2014, Experiment 1	Stroop-trajectory	A newly developed Stroop trajectory task was oriented on either a vertical or horizontal dimension	*Global*. CSE was observed across dimensions
Funes et al., 2010b, Experiments 3 and 4	Spatial Stroop	A Spatial stroop task was varied on horizontal or vertical dimensions with the same (Experiment 3) or a different stimulus set (Experiment 4)	*Global*. CSE was observed across dimensions in both experiments
Kunde and Wühr, 2006, Experiment 1	Prime-target	An arrow prime-target task was presented on either a horizontal or vertical dimension	*Global*. CSE was observed across dimensions
Lee and Cho, 2013, Experiments 1A, 1B, and 4	Simon and Spatial Stroop	The relevant information and conflict type was the same, but the dimension (vertical vs. horizontal) varied in a Simon (Experiment 1A) and Spatial Stroop task (Experiment 1B)	*Specific*. CSE only when the previous and current dimension was the same, even when both dimensions of the Simon task where mapped to the same response (Experiment 4)
Mayr et al., 2003	Flanker	An arrow flanker task was used with either horizontal or vertical arrows	*Specific*. CSE only when the previous and current dimension was the same
**CONFLICT DIMENSIONS**			
Schmidt and Weissman, 2014	Prime-target	The relevant information and conflict type was the same, but the dimension (vertical vs. horizontal) varied in an arrow prime-target (Experiment 1) and word prime-target (Experiment 2) task	*Global*. CSE was observed across dimensions in both experiments
Wühr et al., 2014	Simon	A vertical and horizontal Simon task with shared relevant dimension (color; Experiment 1) or different relevant dimension (shape and color; Experiment 2) were intermixed (Experiment 1)	*Global and Specific* CSE was always observed within dimensions, yet only across dimensions when both tasks shared the relevant dimension
**TASK STRUCTURE, RESPONSE SETS, AND CONTEXT**			
Akçay and Hazeltine, 2008	Simon	Two separate response sets were assigned to either shared or segregated stimuli sets in Experiment 1, 2, and 4, and two segregated stimuli sets were assigned to one response set in Experiment 3	*Global and Specific*. CSE was observed within, but not across segregated stimuli sets assigned to two separate response sets. CES was observed across task sets when either stimuli sets or response sets overlapped
Braem et al., 2011	Simon	Stimulus color determined distinctive response sets (hands and feet) vs. similar response sets (combination of hand responses)	*Specific*. CSE across response sets when similar, but not when distinctive
Braem et al., 2014	Flanker	A flanker task was presented in the context of a visual search experiment where task-irrelevant color could interfere with visual search	*Specific*. CSE was only observed when previous and current task-irrelevant color surrounding the flanker stimulus was the same
Fischer et al., 2010	Simon	Single and double-task contexts were mixed	*Specific*. CSE did not depend on task load, but was only observed within and not across task contexts
Hazeltine et al., 2011, Experiment 2 vs. 3	Prime-target	Two stimuli sets were assigned to one vs. two hands	*Specific*. CSE when assigned to the same, but not when assigned to different response set
Hazeltine et al., 2011, Experiments 1 and 4	Prime-target	One (letters) vs. two (letters and animals) sets of stimuli were used in experiment 1 vs. 4, and stimuli were presented in either visual or auditory modality	*Global and Specific*. CSE only when the preceding and the current stimulus were of the same modality. However, CSE was observed across modalities when two stimuli sets were used
Kiesel et al., 2006	Parity/Magnitude Task	A parity task (press left when odd, right when even) was intermixed with a magnitude task (press left when smaller, right when bigger than five) and conflict originated from incompatible mappings	*Specific*. CSE only when tasks repeated, not when tasks alternated
Kim and Cho, 2014	Flanker	One stimulus set was assigned to four fingers of one hand vs. two times two fingers of both hands	*Specific*. CSE across fingers when assigned to one hand, but not when assigned to two hands
Lee and Cho, 2013, Experiment 2 vs. 3	Simon and Spatial Stroop	The two conflict tasks were assigned to the same, or different hands	*Specific*. CSE across conflict types was observed when the same, but not when a different, response set was used for both tasks
**TASK STRUCTURE, RESPONSE SETS, AND CONTEXT**			
Notebaert and Verguts, 2008	Simon and SNARC	Stimulus color was the relevant dimension in both tasks, or only in one task (and orientation in the other)	*Global and Specific*. CSE was observed across conflict type, but only when task relevant information was the same
Spapé and Hommel, 2008	Color Stroop	Voice gender, irrelevant to the task, was manipulated in an auditory Stroop task	*Specific*. CSE only when previous and current voice gender were the same

### The congruency sequence effect across conflict types

When investigating the scope of conflict adaptation, one of the first research questions that comes to mind is whether or not one conflict type will influence the processing of another—and indeed most relevant studies have investigated just that. We divided these studies into two broad categories, depending on whether or not the different conflict types were combined in a factorial manner (see Egner, 2008). In factorial designs, the two tasks share the same relevant dimension, and the task-irrelevant features are crossed (i.e., both conflict types are combined within each trial). As such, stimuli can be (in)congruent to one of the two irrelevant dimensions, or to both. The second category involves switching designs (Egner, 2008) in which each trial is (in)congruent with respect to only one of the two irrelevant dimensions, and either share the relevant dimension across all trials (i.e., stimulus-switching designs) or not (i.e., task-switching designs). In all of the abovementioned designs, it can be investigated whether conflict adaptation is specific to one conflict type, or transfers across conflict types. Below, we start out with studies that employed a factorial task-crossing design, and then review studies that used task- or stimulus-switching designs.

In a first study, Kunde and Stöcker (2002) factorially combined spatial and temporal Simon conflict. They asked participants to respond by pressing either long or briefly on a left or right key to colored stimuli that were presented left or right from a fixation cross for either a long or short duration. As such, both the correspondence between stimulus and response location (i.e., spatial Simon conflict) and the correspondence between stimulus and response duration (i.e., temporal Simon conflict) were manipulated. The authors did not observe across-conflict CSEs. However, a within-conflict CSE for the temporal Simon task was also not observed. Four years later, Kunde and Wühr (2006) and Wendt et al. (2006) also used factorial designs to study across-conflict CSEs. Kunde and Wühr used a factorial combination of a horizontal Simon task and a spatial prime-target task. Specifically, a prime arrow was presented before the onset of a target arrow and participants had to respond to the direction of this target arrow with a left or right hand button. The direction of prime and target arrows could either correspond or not, and both stimuli were presented at either the left or right hand side of the screen. This way, two types of congruencies were created: a (non)correspondence between the prime and target arrow direction, and a (non)correspondence between the arrow and response location. As expected, Kunde and Wühr (2006) observed a CSE within conflict type: the Simon effect was smaller following an incongruent Simon trial, and the priming effect was smaller following trials with an incongruent prime-target pair. More interestingly, the authors also observed a CSE across conflict types, albeit smaller than for within conflict type. Wendt et al. (2006) used a factorial combination of a Simon task and a flanker task (Experiment 2A), or a Simon and a Stroop task (Experiment 2B). Thus, each trial could be defined by both Simon and flanker conflict by using a task where flanker stimuli were laterally presented, or Simon and Stroop conflict by laterally presenting Stroop stimuli. In both tasks—and in contrast to the study by Kunde and Wühr (2006)—they observed CSEs within conflict type, but not across conflict type.

Several studies followed in the wake of these first seminal observations. Many used similar factorial combinations of two conflict types where each trial could be subject to two types of compatibility effects. For example, Schlaghecken et al. (2011) used a similar design as Kunde and Wühr (2006), but with a centrally, rather than laterally, presented prime. In contrast to Kunde and Wühr, Schlaghecken and colleagues observed no CSE across conflict types. Additionally, instead of using a Simon task as a secondary task as in Wendt et al. (2006), Boy et al. (2010) used a factorial combination of a prime-target task and a flanker task. These authors, too, observed a CSE within, but not across conflict types. Akçay and Hazeltine (2011) followed up more directly on Wendt et al. (2006) by using a similar design where they factorially crossed Simon and flanker conflict whilst controlling for feature repetition effects. Like in the study by Wendt et al. (2006), a CSE was observed within, but not across conflict type (see also Egner et al., 2007). Kim et al. (2012) also found conflict type specific CSEs when using a factorial combination of an arrow and color Stroop task. Last, Kunde et al. (2012) extended this research into the affective domain, by factorially combining a Simon task and an affective interference task. The affective interference task either consisted of a conflict between the relevant and irrelevant stimulus dimension (i.e., affective pictures and affective words, Experiment 1), or a conflict between the relevant response and irrelevant stimulus dimension (i.e., affective verbal responses and smiley faces, Experiment 2). In both experiments Kunde et al. (2012) only observed a CSE within, but not across, conflict types.

Another set of studies explored the transfer of the CSE across conflict types in paradigms where congruency conditions were not factorially crossed within trials, but rather varied across trials (i.e., stimulus- and task-switching designs). First efforts along this line involve a study by Verbruggen et al. (2005), where spatial Stroop trials were intermixed with Simon trials and only CSEs within, but not across, conflict types were observed. Funes et al. replicated this pattern twice with a similar combination between a Simon and spatial Stroop task (Funes et al., 2010a,b), as well as with a spatial Stroop and flanker task (Funes et al., 2010b). Recently, Wühr et al. (2014; Experiment 3) similarly found no evidence for a transfer of the CSE from a manual Simon task to a verbal Stroop task. Last, intermixing Simon and color Stroop trials, Forster and Cho (2014) again demonstrated how CSEs could only be observed within, but not across conflict types.

However, some of these studies did report a CSE across conflict type. Specifically, it has been observed between a Simon and SNARC task (Spatial-Numerical Association of Response Codes; Notebaert and Verguts, 2008), between a number Stroop task and a flanker task or color Stroop task (Fernandez-Duque and Knight, 2008), between vocal flanker and color Stroop task (Freitas et al., 2007; Experiment 2), between joy-stick-based Flanker and spatial Stroop task (Freitas et al., 2007; Experiment 3), and between two different spatial Stroop tasks and an arrow flanker task (Freitas and Clark, 2014). With respect to the study of Fernandez-Duque and Knight (2008), the congruency identity of the previous trial was always cued which renders the design susceptible to more proactive control processes. Also, it must be noted that Rünger et al. (2010) attempted and failed to replicate the results of Fernandez-Duque and Knight (2008). Therefore, the generalizability of this experiment remains to be tested. Moreover, Freitas and Clark (2014) also used a design intermixing a newly developed Stroop trajectory task (to circumvent feature integration and contingency learning confounds) with a factorial combination of a flanker and Simon task (Experiment 3). Notably, while a CSE was observed between the Stroop trajectory task and flanker task, no transfer of the CSE was observed between the Stroop trajectory and Simon task (CSEs between flanker and Simon congruencies were not analyzed).

Finally, two recent studies by Kan et al. (2013) and Kleiman et al. (2014) used a slightly different approach by combining distinctively different tasks with a Stroop or flanker task. Specifically, a recent study by Kan et al. (2013) showed CSEs across tasks by demonstrating how difficult sentence processing experienced in a sentence reading task, or perceptual ambiguity experienced in a perceptual detection task, can decrease the Stroop effect on a subsequent trial. Similarly, Kleiman et al. (2014) demonstrated how flanker congruency on a previous trial can modulate stereotypical biases measured on the current trial. Specifically, stereotypical biases, measured using a gender flanker task (Experiment 1) or race prime-target task (Experiment 2), were only observed following congruent flanker trials, but abolished following incongruent flanker trials.

Taken together, the studies employing factorial combinations of conflict type generally demonstrate that CSEs are conflict type specific, except for the study by Kunde and Wühr (2006), where a CSE between prime-target and Simon effect was still observed (albeit reliably smaller than was the case for within conflict type). On the other hand, studies that investigated the transfer of CSEs across conflict types by using designs where separate trials belong to either one or the other conflict type, have resulted in a more equivocal set of findings.

### The congruency sequence effect across spatial dimensions

A small but substantial number of experiments has been devoted to the detection of CSEs across dimensions but within conflict types. These studies were restricted to spatial congruency effects, where conflict on each trial is induced on either a horizontal or vertical dimension. A first study to investigate this was the study by Mayr et al. (2003), in which an arrow flanker CSE was observed within but not across spatial dimensions. Freitas et al. (2007), however, reported on a reliable CSE across dimensions in a similar setting (Experiment 1), which, they argued, could be due to their shorter stimulus presentation time. Similarly, Kunde and Wühr (2006, Experiment 1) later on demonstrated how the CSE can also be observed across spatial dimensions when administering an arrow prime-target task. Comparable studies followed and CSEs across vertical and horizontal dimensions have been observed in spatial Stroop tasks (Funes et al., 2010b, Experiments 3 and 4), stimulus-response compatibility tasks (Cho et al., 2009), arrow and word prime-target tasks (Schmidt and Weissman, 2014), a Stroop trajectory task (Freitas and Clark, 2014), and the Simon task (Braem et al., 2011; Wühr et al., 2014, Experiment 1). Finally, in addition to Mayr et al. (2003), one more study has failed to observe such CSEs. Specifically, Lee and Cho (2013, Experiments 1A and 1B) did not observe a congruence sequence effect across dimensions when varying dimensions in a Simon (Experiments 1A) or spatial Stroop task (Experiments 2B). Overall, then, with two exceptions (Mayr et al., 2003; Lee and Cho, 2013) it has been consistently demonstrated that CSEs can be observed across vertical and horizontal dimensions within conflict type.

### The impact of task sets on the congruency sequence effect

A third group of studies manipulated specific task parameters to investigate the determinants of CSEs across task sets. According to the definition offered by Schneider and Logan (2014), task sets can be understood as “a set of representations and processes capable of performing a task, including the parameterization of those processes and the identification of their neural substrates” (Schneider and Logan, 2014, p. 29). Importantly, and in strong contrast to some of the work described above, these studies mostly kept conflict type constant, but were interested in whether or not stimulus sets or response sets might co-determine the scope of conflict adaptation. For example, Kiesel et al. (2006) used a parity/magnitude task-switching study in which congruency conditions were created by partially (in)compatible response mappings between both tasks. Using this task, Kiesel et al. (2006) were the first to demonstrate how CSEs are task-specific and thus observed on task repetitions only. Notably, the two tasks used competing response mappings. For example, in the parity task participants had to press left when the number was odd and right when even, whereas in the magnitude task participants had to press left when the number was smaller than five and right when bigger than five. This way, congruent numbers (i.e., “1”) required the same response on both tasks, whereas incongruent numbers (e.g., “2”) did not.

Other studies used either the same, or not necessarily competing, response mappings to investigate the impact of task sets on the specificity of conflict adaptation. In two closely matched conditions, Notebaert and Verguts (2008) demonstrated how their above-mentioned transfer of CSE between Simon and SNARC tasks crucially depended on whether or not both tasks used the same task-relevant information, as the transfer disappeared when both tasks where assigned to different task-relevant information. In a similar vein, Wühr et al. (2014) only showed transfer of the CSE between vertical and horizontal Simon tasks when the relevant dimension was identical between the two tasks (i.e., color), but not when the relevant dimension varied across tasks (i.e., color and shape). Other studies focused on whether or not two tasks use the same response set or not. Braem et al. (2011), for example, demonstrated how a CSE could be observed between a vertical and horizontal Simon task, but only when two highly similar response sets were used (a complex combination requiring both hands for both tasks), and not when both response sets distinctively differed (hand vs. feet). Kim and Cho (2014) observed a similar dependence on response sets in a four-color flanker task where two out of four colors were only presented on odd trials, and the other two on even trials. Specifically, when assigning the four horizontally aligned color buttons to four fingers from one hand, a CSE was observed across colors. However, when the two leftmost buttons were assigned to the left hand (odd trials), and the rightmost buttons to the right hand (even trials), no CSE was observed. In a similar vein, Lee and Cho (2013) showed how a CSE between a spatial Stroop and Simon task could be obtained, but only when the tasks were assigned to the same response hand (Experiments 2 vs. 3). Again using a Simon task, Akçay and Hazeltine (2008) likewise demonstrated how the CSE could not be observed when two segregated stimuli sets were assigned to two separate response sets. However, whenever either the stimuli sets or the response sets overlapped, a CSE across conditions was found. In a similar vein, using a prime-target paradigm, Hazeltine et al. (2011) demonstrated how two stimuli sets assigned to either one or two hands only showed an across-set CSE when both sets were assigned to one hand (Experiments 2 vs. 3). Overall, these studies indicate that the partitioning of particular stimuli and response sets within a certain task can be a sufficient condition to observe set-specific CSEs.

### Contextual task-irrelevant factors and the congruency sequence effect

A final set of studies investigated the CSE as a function of task-irrelevant contextual factors. Most notably, Spapé and Hommel (2008) demonstrated how the Stroop CSE is sensitive to voice gender in an auditory Stroop task. In their Stroop task, color detection could be facilitated or hampered by the auditory presentation of congruent or incongruent words spoken by male or female voices. Interestingly, CSEs were only observed when voice gender repeated, but not when voice gender alternated. In a similar vein, Hazeltine et al. (2011) demonstrated how the CSE depended on whether or not the previous and current stimuli were of the same modality (visual or auditory, Experiment 1). However, when increasing stimulus set heterogeneity by introducing a second different category of stimuli, this modality-specificity of the CSE disappeared (Experiment 4). Investigating the impact of task load on the CSE, Fischer et al. (2010) observed how the CSE was dependent on whether both the current and previous trial were presented in both dual task or single task conditions, but not when alternating between them. The authors concluded that task context, rather than task load, is a crucial determinant in bringing about the CSE. Last, Braem et al. (2014) recently observed how a flanker CSE depends on whether or not a surrounding shape is presented in the same, or rather in an alternative color as the previous trial. By pairing a visual search task with a flanker task (i.e., participants had to search a unique shape out of six shapes and respond to the flanker task presented within that shape), these authors demonstrated that only under conditions where task-irrelevant shape color repeated, a congruence sequence effect occurred. Together, these experiments all seem to suggest that the repetition or alternation of task-irrelevant contextual salient features can codetermine whether or not a CSE will occur.

## Critical analysis

On the basis of our review we can identify two important factors determining the specificity of conflict adaptation: conflict type and context-similarity. When it comes to conflict type, we agree with Egner's (2008) review that in full-factorial designs there is abundant evidence that the CSE appears to be conflict type-specific (Egner, 2008). Only Kunde and Wühr (2006) observed a reliable, albeit smaller, CSE across congruency conditions, which, according to Egner (2008), was most likely due to the high similarity between the two conflict types. Consistently, when Schlaghecken et al. (2011) attempted to replicate Kunde and Wühr (2006) with a centrally (rather than laterally) presented prime, they did not observe a CSE across conflict types.

A second line of research used non-factorial designs. Note that Egner (2008) argued against using such designs because they are often confounded with switch costs and do not allow the researcher to investigate if both conflict types are independent (additive) or not. Importantly, Egner (2008) was only interested in the conflict-specificity of the CSE, whereas we, in our review, are interested in the scope of conflict adaptation more generally (across various conditions). Therefore, we consider switch costs a second, informative symptom of the same phenomenon that might explain the absence of a transfer of the CSE across conditions: participants represented both conflict types as deriving from two different task sets (see below). For this reason, we will also discuss these studies, but treat them separately as studies indexing the impact of task sets or context, rather than conflict type. Although some of these studies' main intention was to investigate the conflict-specificity, the impact of conflict type cannot be disentangled from task set or task context. Therefore, these studies do not allow to make more specific inferences about conflict-specificity (Egner, 2008).

Interestingly, despite the fact that most studies using non-factorial designs found task-specific CSEs, some found across task CSEs. Most of these across-task—and thus relatively domain-general—CSE observations can be ascribed to using either similar conflict types (e.g., Fernandez-Duque and Knight, 2008; Notebaert and Verguts, 2008; Freitas and Clark, 2014) and/or very similar response mappings (e.g., Freitas et al., 2007; Notebaert and Verguts, 2008). Indeed, when using different conflict types (Freitas and Clark, 2014), or different response mappings (Notebaert and Verguts, 2008), some of these studies reported task-specific CSEs as well. Additionally, Rünger et al. (2010) attempted and failed to replicate the results of Fernandez-Duque and Knight (2008) so there might be need for further replication studies (see suggestions for replication endeavors below). However, it is not our aim to refute these domain-general CSE observations as methodologically flawed. In fact, such observations might sometimes be obscured by the lack of statistical power to observe more subtle across-domain CSEs. Moreover, as we will argue below, these observations might be expected in cases where both task sets are not interfering with each other.

In contrast to the full-factorial designs described above, we cannot conclude on whether or not these studies evidence the conflict specificity of the CSE. However, at the very least these studies do suggest that task structure can determine the specificity of CSE. This idea that task sets might play an important role in determining the scope of the CSE is not new. Indeed, as reviewed above, Hazeltine and colleagues (Akçay and Hazeltine, 2008; Hazeltine et al., 2011) clearly stressed the role of task sets and task boundaries in bringing about CSEs. Recent evidence seems to support this hypothesis. An increasing number of papers have demonstrated this importance of stimulus and response sets by demonstrating how CSEs across task sets do not occur when both sets are clearly distinguishable and thus perceived as different task sets, especially when both sets have conflicting response mappings. Interestingly, this does not necessarily depend on the complexity of the task, which naturally increases with using multiple stimuli sets. In fact, Hazeltine and colleagues demonstrated how increasing stimulus set size and modality to the extent that it is not longer beneficial to dissociate the different sets, allows for across-modality and across-set CSEs to occur (Hazeltine et al., 2011, Experiment 4). We believe this role of task sets remains an under-investigated aspect of conflict adaptation, and we will offer some suggestions below as to how the implementation and impact of such instructed task sets can be further investigated.

The adaptation-by-binding theory offers a computational implementation of conflict adaptation. It states that upon conflict detection, a general Hebbian learning signal (“now print”) is sent throughout the brain to strengthen all active ongoing associations. Task set representations are activated by task demand units which can, just like simple stimulus or response units, be strengthened following conflict detection. Therefore, this theory can account for the susceptibility of CSEs to task set boundaries. However, it actually goes one step further and predicts that also task-irrelevant features can influence the scope of conflict adaptation. In fact, everything that is salient and/or systematically co-activated can be picked up by this Hebbian learning mechanism and incorporated in the strengthening of associations. This way, we can expect that if a salient event occurs in the temporal vicinity of conflict detection, task associations might become temporarily associated with this stimulus feature or event and CSEs can therefore be specific to the repetition of this event. Indeed, as reviewed above, a number of studies have observed such context-specific CSEs. In some of these studies, this effect was not anticipated (e.g., Fischer et al., 2010), so there is clear room for further systematic investigations on the impact of such contextual features.

## Proposed synthesis

**Figure 1:**
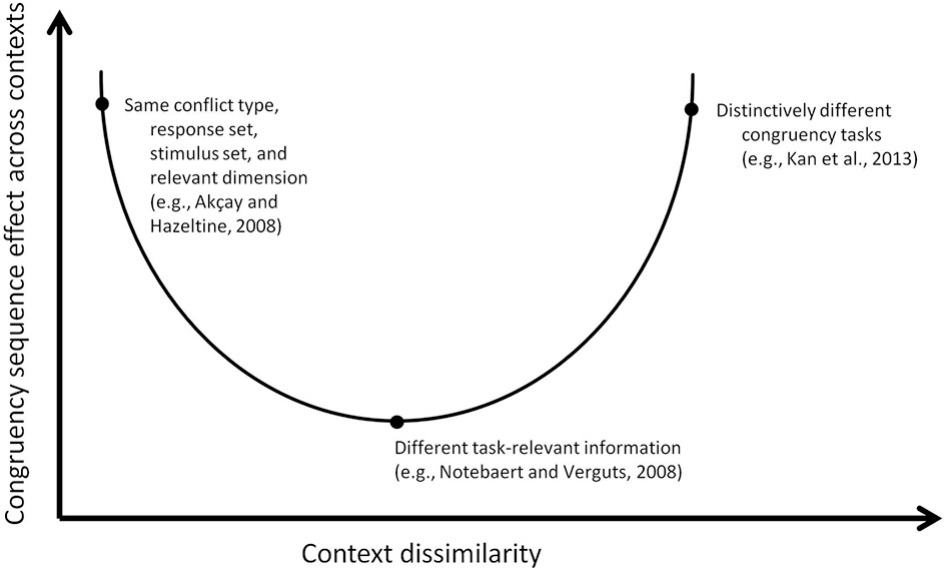
**Abstract depiction of the hypothesized u-shaped relation between context (dis)similarity and congruency sequence effects across contexts**. As per example, three different empirical studies are displayed along the function.

Taken together, these stimulus-, task-, or context-switching designs might be integrated by the general principle that conflict adaptation is highly specific to the context—where we should understand context in its broadest sense to cover the impact of both (instructed) task-relevant features (including, for example, S-R mappings) as well as task-irrelevant (but salient) features. However, how then should we understand the few studies that seem to refute context-specificity, such as those of Kan et al. (2013) and Kleiman et al. (2014) which demonstrated that CSEs can be observed across very different task sets? Therefore, we propose the working hypothesis that transfer of CSEs can be observed across contextual features (both task-relevant and -irrelevant) as long as these features are simultaneously and actively maintained (in working memory). Studies in which two contexts are used that would substantially interfere with each other when they are both actively maintained, will result in strategies where only one context is active at any time such that transfer is prevented. This fits nicely with interference based models of working memory as developed by Oberauer and colleagues (Oberauer and Kliegl, 2006; Oberauer et al., 2012) where working memory capacity restrictions arise from interference, rather than, for example, limited resources. Similarly, Oberauer et al. (2012) model suggests that interference in working memory will be greater when (task) features overlap or belong to similar categories. Therefore, two contexts that are sufficiently different such that simultaneous maintenance is possible without much interference (cf. Kan et al., 2013; Kleiman et al., 2014), may result in transfer across (very different) contexts. Hence, whereas one would predict a linear relation between context similarity and the chance of observing transfer of the CSE from the idea that task sets determine the scope of conflict adaptation (Hazeltine et al., 2011), we predict a U-shaped relationship (see Figure 1): transfer is observed whenever two contexts (task-relevant and -irrelevant) are either very similar or sufficiently dissimilar to prevent interference.

Importantly, this idea is not entirely new. Hazeltine and colleagues made a similar observation when discussing the findings of Freitas et al. (2007) who did observe a CSE across task sets. Specifically, they argued that because Freitas and colleagues increased the heterogeneity within their tasks, and switched the tasks randomly, the salience of the task boundaries was reduced, allowing CSEs to occur across task sets (Hazeltine et al., 2011). This fits with our proposal that *interfering contexts* or *interfering tasks* rather than *context or task similarity* are the key factor in determining the scope of conflict adaptation. Interestingly, this might also explain why factorial combination studies mostly observe conflict-specific CSEs while similar investigations using stimulus-switching designs have sometimes observed across-conflict CSEs. Whereas the former closely intermix different congruency conditions, the latter dissociate both congruency conditions to the extent that they can again either be perceived as one task set, especially when they share task-relevant information (Notebaert and Verguts, 2008), or as two non-interfering task sets, that can easily be maintained in parallel in working memory.

This idea is also compatible with the computational model of Verguts and Notebaert (2008, 2009). In their adaptation by binding theory, Verguts and Notebaert propose that following conflict detection, a general Hebbian learning signal is sent throughout the brain that reinforces all active and ongoing associations. Importantly, although the model is blind to what is task-relevant or not, task-relevant associations are mostly the ones that are strengthened since these are the most active during conflict-resolution. Whenever tasks are defined on the basis of features (e.g., S-R mappings) that are mutually interfering, the result may be that at each moment in time (i.e., each trial) only one of the two tasks can be actively maintained—and thus that binding processes underlying adaptation are specific to one but not the other task (no “transfer” between tasks). Now, in such designs where interfering associations (e.g., from interfering task sets) have to be suppressed to ensure successful conflict resolution, we can predict that these task sets will not benefit from this Hebbian learning signal, and domain-specific CSEs will be observed (halfway the U-shaped function on Figure 1). However, when both task sets are very compatible either because they are highly similar (left hand side of the U-shaped distribution on Figure 1), or because they are highly distinctive and can be simultaneously kept available in working memory (right hand side of the U-shaped distribution on Figure 1), domain-general CSEs might occur.

Lastly, we would like to add that memory-based theories (Hommel et al., 2004; Schmidt, 2013) should not—as is currently the case—be seen as theoretical alternatives to conflict adaptation theories (for similar arguments, see Spapé and Hommel, 2008; Verguts and Notebaert, 2008; Braem et al., 2011; Hazeltine et al., 2011; Jimenez and Méndez, 2014). In fact, these theories and their predictions concerning the specificity of CSEs are largely compatible to the above-made predictions and observations. For example, using their theory of event codes, Hommel et al. (2004) proposed that the CSE can be understood in terms of slower partial repetitions vs. faster complete repetitions or alternations of event files (see above). This view can easily be extended to task sets or contexts. CSEs without feature repetitions have now been demonstrated (e.g., Duthoo and Notebaert, 2012), but it is still possible that (latent) partial repetitions of certain task sets can prevent CSEs from occurring across task sets. In fact, this idea is compatible with our proposed U-shaped function between task-similarity and CSEs across tasks: complete repetitions (of largely overlapping task sets) or complete alternations (of distinctively different task sets) can allow for across-task CSEs to occur, while partial repetitions (task sets partially sharing features or resources) are more demanding for working memory and therefore induce task-specific CSEs. Interestingly, these learning theories motivate us to further pursue the research question how exactly task structures are implemented, remembered, and dealt with, while simultaneously investigating the impact of contextual features and associative learning. However, although learning theories as such, we believe, offer important theoretical insights, and need to be taken into account when considering modulations of cognitive control, they do need to be controlled for when using the CSE as a metric of conflict adaptation. For example, while the theory of event coding (Hommel, 2004; Hommel et al., 2004) is consistent with our proposed U-shaped function between context-similarity and CSEs across contexts, it does not account for observations showing CSEs devoid of feature integration, as it does not acknowledge a role for congruency identity of the previous trial as an important determinant for the CSE. Importantly, such CSEs devoid of feature integration and contingency learning have been demonstrated (for a comprehensive review in this same issue, see Duthoo et al., 2014), but only recently. As a result, only a small number of the above-mentioned studies controlled for both feature integration and contingency learning (Freitas and Clark, 2014; Kim and Cho, 2014; Schmidt and Weissman, 2014). These paradigms should be taken as guides for further research on the specificity (and other modulations) of conflict adaptation. In fact, such experiments are much needed as it is currently unclear to which extent earlier studies on the CSE (and their modulations) could have been attributed to their confound with feature integration or contingency learning.

## Conclusions, challenges, and guidelines

In sum, we reviewed the most prominent theories, and, to our knowledge, all studies that investigated the specificity of conflict adaptation. We identified a number of elements that can determine the scope of conflict adaptation. In fact, in line with the reviewed theories, conflict type (Egner, 2008), task set (Hazeltine et al., 2011), and context (Verguts and Notebaert, 2009), all seem to play a central role in determining whether or not across-condition CSEs will occur. In contrast, conflict dimension (vertical vs. horizontal) did not appear to be a crucial factor (but see Mayr et al., 2003; Lee and Cho, 2013). To account for the current set of data, we proposed a U-shaped function between context similarity and cross-condition conflict adaptation. Therein, we stress the role of task sets and whether or not they can be simultaneously activated in working memory. We believe there are still a number of challenges ahead, opening up new opportunities for further research. Therefore, we will end by identifying some of those challenges and offer a number of tentative guidelines on how one might tackle them.

First, we illustrated how the current state of the art in conflict adaptation research and its specificity can be understood in terms of a U-shaped relation between transfer of the CSE and context similarity. Importantly, whenever both task sets can be simultaneously updated because they are either highly similar or distinctively different and non-interfering, adaptation across tasks and conditions can be observed. We believe this hypothesis is testable or falsifiable by using a design where task sets or conditions are parametrically dissociated. However, in setting up such a design it will be important to develop a paradigm where the task-rules are not overly complicated (participants should still be able to keep both tasks in working memory).

Second, we re-emphasize the importance of taking into account task sets when investigating the specificity of conflict adaptation (Hazeltine et al., 2011). In this respect, a promising new research field on task instructions has developed a number of interesting paradigms that allow us to test the effects of task sets whilst controlling for the repetition or alternation of more low-level stimulus or response feature characteristics. For example, Dreisbach et al. (2007) elegantly demonstrated how it is possible to introduce a difference in task sets by mere instructions, without having to manipulate stimulus features or stimulus-response mappings. Specifically, in their study, Dreisbach et al. (2007; see also Dreisbach and Haider, 2008, 2009) trained participants at certain stimulus-response (S-R) rules between eight stimuli and two responses after which they could either receive, or not receive, an overarching rule that is able to categorize the same S-R rules as belonging to one out of two task sets. Interestingly, while both groups performed sufficiently well at the S-R rules, performance in the late-informed task set group was worsened, relative to the uninformed group, as evidenced by task-switch costs following task-rule instructions. Clearly, this implementation of task sets introduced new task boundaries that interfered with switching between the two groups of stimuli. Importantly, paradigms like these can allow us to investigate the impact of task sets on conflict adaptation, without having to create different stimuli. Moreover, these studies allow us to investigate how exactly task sets are implemented as this remains an under-investigated issue in the cognitive control literature (see also Everaert et al., 2014; Liefooghe et al., 2012).

Third, we discussed a number of recent studies that demonstrated the impact of salient task-irrelevant uninstructed features on the specificity of conflict adaptation. However, these studies are still relatively scarce, and a systematic investigation of these effects seems warranted. Moreover, whereas theories predicting these effects (Verguts and Notebaert, 2008) seem to emphasize these kind of bottom-up effects, others have emphasized top-down effects (Hazeltine et al., 2011). We have stressed the importance of both instructed task sets as well as the impact of these contextual features. Therefore, an interesting challenge remains the investigation of how the impact of task-irrelevant uninstructed features might still differ from the impact of instructed task sets on conflict adaptation, as well as how they interact. We argued that both can co-determine the scope of conflict adaptation. However, future research should offer a more nuanced view by unraveling the complex interplay between the effects of (top-down) task sets vs. (bottom-up) context on conflict adaptation.

Lastly, researchers should engage in trying to test the impact of specificity with paradigms that are free of feature integration or contingency learning confounds (Freitas and Clark, 2014; Kim and Cho, 2014; Schmidt and Weissman, 2014; Weissman et al., 2014). This is non-trivial, as most measures of the CSE thus far can be alternatively explained by low-level memory effects. In another review article in this same issue, we outline the specific problems that one needs to take into account, and outline a number of guidelines on how researchers can develop the appropriate paradigms (Duthoo et al., 2014). However, this need for dissociating learning effects from CSEs is purely methodological, and should not be mistaken for a motivation to theoretically distance those from conflict adaptation research. In fact, here, as well as in earlier works (Spapé and Hommel, 2008; Verguts and Notebaert, 2008; Braem et al., 2011; Hazeltine et al., 2011; Jimenez and Méndez, 2014), it has been argued that memory effects and associative learning can offer important insights in the underlying mechanisms and dynamics of conflict adaptation.

### Conflict of interest statement

The authors declare that the research was conducted in the absence of any commercial or financial relationships that could be construed as a potential conflict of interest.
